# Hemolytic uremic syndrome caused by Shiga toxin-producing *Escherichia coli* in a renal transplant recipient case report

**DOI:** 10.1590/2175-8239-JBN-2020-0048

**Published:** 2020-11-11

**Authors:** John Fredy Nieto-Rios, Monica Zuluaga-Quintero, Julio Cesar Valencia-Maturana, Diana Carolina Bello-Marquez, Arbey Aristizabal-Alzate, Gustavo Adolfo Zuluaga-Valencia, Lina Maria Serna-Higuita, Luis Fernando Arias

**Affiliations:** 1 Hospital Pablo Tobón Uribe, Department of Nephrology and Kidney Transplant, Medellín, Colombia.; 2 University of Antioquia, Department of Internal Medicine and Pediatrics, Medellin Colombia.; 3 Hospital Pablo Tobón Uribe, Department of Internal Medicine, Medellin, Colombia.; 4 Eberhard Karls University, Institute of Clinical Epidemiology and Biometrics, Tuebingen, Germany.; 5 University of Antioquia, Department of Pathology, Medellin, Colombia.

**Keywords:** Thrombotic Microangiopathies, Hemolytic-Uremic Syndrome, Shiga Toxin, Kidney Transplantation, ADAMTS13 Protein, Complement Pathway, Alternative., Microangiopatias Trombóticas, Síndrome Hemolítico-Urêmica, Toxina Shiga, Transplante de Rim, Proteína ADAMTS13, Via Alternativa do Complemento.

## Abstract

Thrombotic microangiopathies are disorders characterized by nonimmune microangiopathic hemolytic anemia, thrombocytopenia, and multi-systemic failure. They are classified as thrombotic thrombocytopenic purpura, atypical hemolytic-uremic syndrome, and typical hemolytic uremic syndrome. The latter is associated with intestinal infections by Shiga toxin-producing bacteria. Typical hemolytic uremic syndrome in adults is an extremely rare condition, characterized by high morbidity and mortality. It has been seldom described in solid organ transplant recipients. Here is presented the case of a kidney transplant recipient who had typical hemolytic uremic syndrome with multisystem commitment, refractory to management and with a fatal outcome.

## Resumo

Microangiopatias trombóticas são distúrbios caracterizados por anemia hemolítica microangiopática não imune, trombocitopenia e insuficiência multissistêmica. Elas são classificadas como púrpura trombocitopênica trombótica, síndrome hemolítico-urêmica atípica e síndrome urêmica hemolítica típica. Essa última está associada a infecções intestinais por bactérias produtoras da toxina Shiga. A síndrome hemolítica urêmica típica em adultos é uma condição extremamente rara, caracterizada por alta morbimortalidade. Esta é raramente descrita em receptores de transplantes de órgãos sólidos. Apresentamos aqui o caso de um receptor de transplante renal que apresentava síndrome hemolítico-urêmica típica com comprometimento multissistêmico, refratário ao tratamento, e com desfecho fatal.

## Introduction

Hemolytic uremic syndrome (HUS) is a rare disease but with high morbidity and mortality^1^. It is characterized by the presentation of nonimmune microangiopathic hemolytic anemia, thrombocytopenia, and multi-system failure with mainly renal involvement. Two clinical entities are currently described: hemolytic uremic syndrome caused by Shiga toxin-producing *Escherichia coli* (STEC-HUS), also known as typical HUS, and atypical HUS (aHUS), which can be associated with immunological, infectious, neoplastic, toxic, hemodynamic, gestational causes, etc. (secondary aHUS), or due to an alteration in the regulation of the alternative complement pathway (primary aHUS)^2^^,^ 3.

In STEC-HUS, the Shiga toxin causes direct endothelial damage, with increased production of pro-inflammatory cytokines, increasing the risk of thrombosis with damage to different organs, mainly the kidneys. Additionally, this toxin activates the alternative pathway of the complement system, amplifying the inflammatory response. STEC-HUS is initially manifested by abdominal pain, vomiting, and diarrhea, and within a period of 5 to 10 days after the onset of symptoms, renal function disorder, non-immune hemolytic anemia, and thrombocytopenia occur. The degree of renal involvement varies from the presence of hematuria and proteinuria to the development of severe acute renal injury, which requires renal replacement therapy in up to 50% of patients; neurological compromise may also present in about 25% of cases, which is associated with high mortality^1^.

STEC-HUS has been described in 6 to 9% of cases of gastrointestinal infection by certain entero-invasive strains of *E. coli* 3 representing an important cause of acute kidney injury in children, with an incidence of 6 cases per 100,000. However, there are few reports in adults, with a reported incidence of 2 cases per 100,000, being even more rare in patients with both solid organ and bone marrow transplantation. For this reason, this disease is little suspected in transplant recipients, which leads to a late diagnosis, introduction of treatment in advanced stages, and a worse prognosis^4^. Here is presented the case of a kidney transplant recipient who developed STEC-HUS with multi-system failure, without response to the prescribed management, and with a fatal outcome.

## Clinical Case Description

A 63-year-old Caucasian man with end-stage renal disease secondary to diabetic nephropathy, was on maintenance hemodialysis. His comorbidities included type 2 diabetes mellitus and hypertension since 2005. Five months after starting dialysis, the patient underwent a kidney transplant from a standard deceased donor, aged 57 years at decease time, compatibility 1 DR - 1A, cold ischemia time 19 hours, immediate graft function (November 12, 2017). He received induction with a single dose of 100 mg of thymoglobulin, and 500 mg-day pulses of methylprednisolone for 3 days. He was maintained with 3 mg-day tacrolimus XL, for levels 5 to 10 ng/mL, 1440 mg-day sodium mycophenolate, and 10 mg-day prednisone. The patient reached a baseline creatinine of 2.36 mg/dL a week later, 1.4 mg/dL a month later, and 1.2 mg/dL at three months. He received prophylaxis with 960 mg trimethoprim sulfamethoxazole three times a week and valganciclovir adjusted to his renal function for 100 days. On March 25, 2018, he was examined for 3 days of evolution of asthenia, adinamia, hyporexia, sensory disturbance, oliguria, and edema. He reported scarce liquid non-bleeding diarrhea and abdominal pain starting a week earlier. On physical examination, he was found dehydrated, with tachycardia (heart rate 98 per minute), normal blood pressure values (127/80 mmHg), respiratory rate 15 per minute, normal temperature, and non-painful soft abdomen, without irritation, with increased peristalsis. On neurological examination, he presented with drowsiness, disorientation in time, without meningeal signs. In the initial tests, documented leukocytosis with neutrophilia, anemia, thrombocytopenia, elevated protein C reactive values, and azotemia were detected (see Table 1). The initial CT scan of the skull was normal. A lumbar puncture was performed, and cerebrospinal fluid (CSF) cells count was 0 per mm^2^. CSF protein and glucose levels was normal (glucose 92 mg/dL with blood sugar 110 mg/dL). CSF Gram, acid-fast bacilli, and fungal smears and cultures were all negative. Polymerase chain reactions (PCR) for central nervous system pathogens were also negative (see Table 1). Due to neurological, renal, and hematological involvement, an initial diagnosis of gastrointestinal sepsis was made. Antibiotic treatment with piperacillin-tazobactam was initiated, prior to taking microbial cultures. A day after hospitalization, he presented greater neurological deterioration, progressing to coma, with the need for orotracheal intubation; he also developed oliguria. A workup was performed to rule out other diseases; the values for coagulation studies and fibrinogen were within normal limits and the disseminated intravascular coagulation (DIC) score was negative, which ruled out the diagnosis of DIC. The ADAMTS13 activity was measured three days after the admission, which was normal (82.8%).

**Table 1:** Laboratory tests results

Hematologic profile		Microbiological tests		Blood chemistry tests	
Hemoglobin (g/dL)	9.1	EHEC Shiga Toxin in stools. DNA detector (PCR)	positive	Sodium (mEq/L)	142
Hematocrit (%)	26.8	Stool culture	negative	Chloride (mEq/L)	118
Leukocytes (mm^3^)	12100	Urine cultures	negative	Potassium (mEq/L)	4.76
Platelets (mm^3^)	18000	Blood cultures	negative	Calcium (mg/dL)	8.2
Neutrophils (%)	96	Cerebrospinal fluid culture	negative	HCO3 (mmol/L)	18
Reticulocytes (%)	3	Serology for dengue	negative	Lactate (mmol/L)	0.7
ESR (mm/hour)	21	Coccidia in stools	negative	Cai: (mmol/L)	1.25
PCR (mg/dL)	4.53	Viral load test for CMV, serum	negative	AP (U/L)	66
TP (sec)	11.7	Viral load test for E. Barr, serum	negative	UA (mg/dL)	6.0
TPT (sec)	26.4	Viral load test for BK virus, serum	negative	CK (U/L)	111
Schistocytes	++	Latex agglutination test for *Cryptococcal* antigen, serum	negative	Albumin (g/dL)	3.2
Direct coombs test	Negative	Serology for mycoplasma	negative	Creatinine (mg/dL)	3.04
LDH (U/L)	2674	**PCR panel for CNS pathogens**		BUN (mg/dL)	103
Haptoglobin (mg/dL)	< 8	*Escherichia coli* K1	negative	AST (U/L)	115
Dimer D (ng/ml)	9472	*Haemophilus influenzae*	negative	ALT (U/L)	31
Fibrinogen (mg/dL)	436	*Listeria monocytogenes*	negative	BD (mg/dL)	0.96
ADAMTS-13 (%)	82.8	*Neisseria meningitidis*	negative	GGT (U/L)	25
Ferritin (ng/mL)	893	*Streptococcus agalactiae*	negative	C3 (mg/dL)	120
**Urine test**		*Streptococcus pneumonie*	negative	C4 (mg/dL)	18
Proteinuria (mg/dL)	500	Cytomegalovirus (CMV)	negative		
Glycosuria (mg/dL)	50	*Herpes simplex virus* 1 and 2	negative		
Leukocytes (HPF)	6-10	*Human herpesvirus* 6	negative		
Erythrocytes (HPF)	6-10	*Varicella zoster virus*	negative		
24-hour Proteinuria (gr/day)	6.1	*Cryptococcus neoformans*	negative		

PBS: Peripheral blood smear; sec: seconds; eGFR: estimated Glomerular Filtration Rate; ESR: erythrocyte sedimentation rate, PCR: C-reactive protein; PT: Prothrombin time; TPT: Thromboplastin time; EHEC: Enterohemorrhagic Escherichia coli; CMV: cytomegalovirus; Cai: ionized calcium, AST: aspartate transaminase; ALT: alanine transaminase. BD: direct bilirubin test; AP: alkaline phosphatase; GGT: gamma glutamyl transpeptidase; UA: uric acid; HPF: high power field; LDH: lactate dehydrogenase; CK: creatin-Phosphokinase; C3 and C4: Seric Complement C3 and C4.

**Figure 1:**
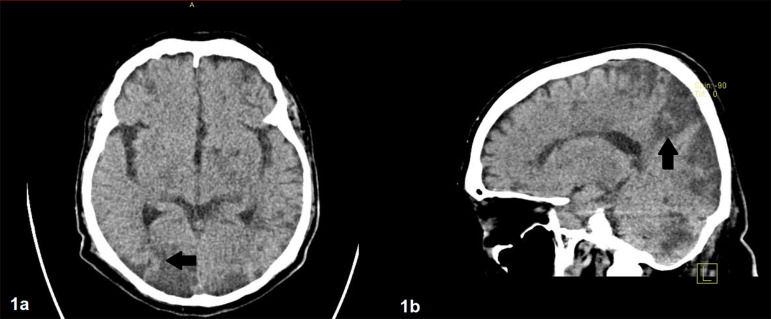
Brain CT showing extensive cerebral infarctions. Hypodensities of the white and gray matter in both cerebral hemispheres are observed in bordering territories, with hypodensities in the posterior fossa and brain stem (arrows). There are no subdural or epidural hematoma, nor subarachnoid hemorrhage. The size and morphology of the ventricular system are normal.

**Figure 2:**
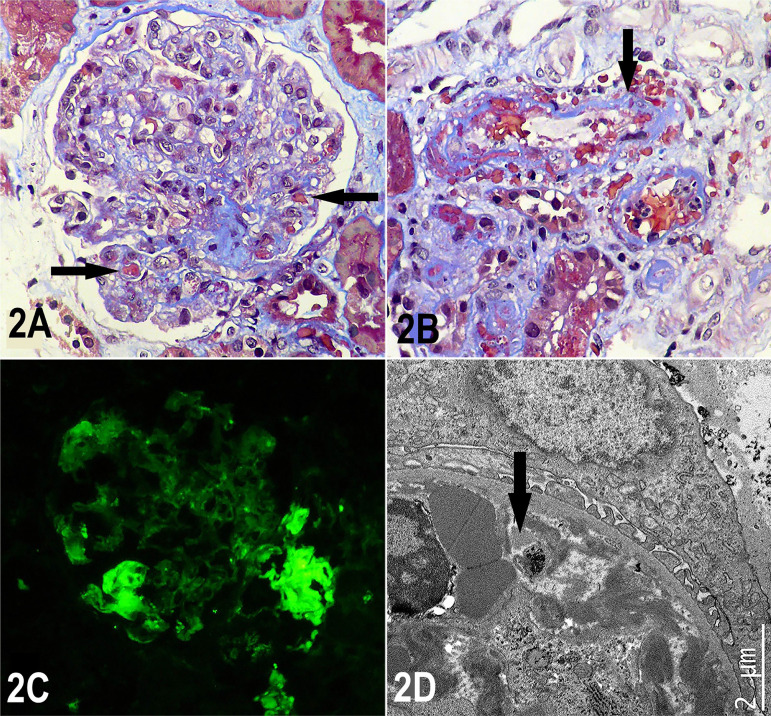
Histological findings in renal biopsy. Figure 2A: Glomerulus with solidified appearance, with marked decrease in capillary diameters due to endothelial edema and some intra-capillary thrombi (arrows). Masson's Trichrome, X400. 2B: Small diameter artery with non-occluding thrombus, erythrocyte extravasation, and wall necrosis (arrow). Masson's Trichrome, X400. 2C: Direct immunofluorescence for fibrinogen demonstrating positivity in some glomerular capillaries (thrombi), X400. 2D: At electron microscopy can be observed how the diameter of a capillary blood vessel is blocked/narrowed by thrombi, with fibrin and fragmentation of erythrocytes and platelets (arrow); podocytes and basal membranes show normal appearance.

Laboratory tests showed an increase in lactic dehydrogenase, consumed haptoglobin, increased thrombocytopenia and anemia, schistocytosis, and elevated serum creatinine (Table 1). A new skull tomography was performed, which reported multiple cerebral infarctions (Figure 1). A clinical diagnosis of thrombotic microangiopathy (TMA) with multi-system compromise was made, which is why tacrolimus was withdrawn, plasma changes were indicated, as well as transfusion support, and hemodialysis was initiated. In addition, a kidney biopsy was performed, which showed thickening of the glomerular capillary walls, endothelial edema, and presence of some micro-thrombi occluding capillary light, plus erythrocyte fragmentation. In some small-sized arteries and arterioles, recent thrombi were observed, with wall necrosis and erythrocyte extravasation, foci of interstitial hemorrhage, and acute tubular damage. Histological findings compatible with rejection were not found, the immuno-staining for C4d was negative (Figure 2), and donor specific antibodies were negative, therefore cellular and/or humoral graft rejection were ruled out. Tests for viral infections were negative. Seven days after admission, the Shiga toxin in stool testing, performed three days after the hospitalization, resulted positive and, then, STEC-HUS was finally diagnosed. It was decided to continue without tacrolimus. Mycophenolate therapy was also withdrawn, leaving only prednisolone at 15 mg/day. The patient did not respond to supportive treatment and presented deterioration and progression to multi-system failure with pulmonary, renal, and cardiac involvement (acute myocardial infarction) and severe extension of neurological infarcts with an unrecoverable deep coma. The patient died 15 days after admission.

## Discussion

This case report describes a kidney transplant recipient who presented STEC-HUS with neurological involvement which proved fatal. According to our knowledge, this the third case reported in the literature.

HUS is a serious complication that can occur after renal transplantation. Epidemiological studies report an incidence of 5.6 episodes per 1000 person-years, and with a 50% mortality three years after diagnosis^5^. The main differential diagnosis of HUS is thrombotic thrombocytopenic purpura (TTP) associated with decreased (below 10%) activity of the ADAMTS13 enzyme, which is related to autoimmunity (antibodies against ADAMTS13) or rarely to mutations of this enzyme^2^^,^ 6.

Post-transplant HUS is classified into two categories: recurrent HUS, in which the same disease process that manifested as TMA in the native kidney develops again in the allograft, and whose primary example is the primary aHUS, and *de novo* HUS after transplantation, which develops for the first time in a patient who had never had evidence of TMA before transplantation, being most often associated with secondary causes (secondary HUS)^7^, such as toxicity to calcineurin or mTOR inhibitors, acute antibody-mediated rejection, opportunistic infections (viruses, fungi, mycobacteria), etc^5^. STEC-HUS (associated with Shiga toxin) is an extremely rare complication in this group of patients^6^.

In the reported case, the patient presented a *de novo* TMA associated with STEC-HUS, with hematological, neurological, renal, pulmonary, and cardiovascular compromise, and with a fatal outcome. These complications have been less reported in cases of HUS in non-transplanted populations^2^^,^ 3; thus, we consider that there may be underreporting, whether because the entity is not suspected or because of the severity of the clinical course, a late diagnosis is made. Therefore, it is proposed the hypothesis that the typical HUS (STEC-HUS) in transplant recipients has a more aggressive behavior, which could be explained by the immunosuppressive therapy received after transplantation.

The treatment of STEC-HUS is not fully defined in the literature. In children, it is based on support management, with dialysis, transfusions, and antihypertensive drugs, and in adults, in addition to the above, some authors propose plasma exchange, others, eculizumab, without solid evidence in favor of one or another therapy^7^^-^ 10. In relation to post-transplant STEC-HUS, a case has been described in a patient with lung transplant who had a serious neurological compromise in response to management with plasma exchange^11^. Another report describes a patient with a bone marrow transplant who presented neurological symptoms and deterioration of renal function, and who received management with eculizumab with improvement of neurological symptoms, as well as stabilization of renal function at 7 months of follow-up^4^. Ville et al. described five patients with solid organ transplants who presented STEC-HUS, of which two were kidney transplants, and in whom eculizumab was used but with inconclusive results^12^. However, eculizumab is not currently recommended for the treatment of STEC-HUS in any population, as there is no evidence of benefit.

In conclusion, TMA is a rare but very serious complication in kidney transplant recipients. We suggest that those with gastrointestinal symptoms should be studied for STEC-HUS by performing Shiga toxin test in stools and/or looking for enteroinvasive *E. coli*. Since the specific treatment of this entity has not yet been elucidated, early multidisciplinary support management could improve outcomes. The role of therapies such as eculizumab should be evaluated in the long term.
